# Regulation of the dystrophin-associated glycoprotein complex composition by the metabolic properties of muscle fibres

**DOI:** 10.1038/s41598-019-39532-4

**Published:** 2019-02-26

**Authors:** Saleh Omairi, Kwan-Leong Hau, Henry Collins-Hooper, Charlotte Scott, Sakthivel Vaiyapuri, Silvia Torelli, Federica Montanaro, Antonios Matsakas, Ketan Patel

**Affiliations:** 1 School of Biological Sciences, University of Reading, Reading, UK; 2 College of Medicine, Wasit University, Kut, Iraq; 3 UCL Great Ormond Street Institute of Child Health, Developmental Neurosciences, Programme, London, UK; 4 NIHR Great Ormond Street Hospital Biomedical Research Centre, 30 Guilford Street, WC1N 1EH London, UK; 5 School of Pharmacy, University of Reading, Reading, UK; 6 Molecular Physiology Laboratory, Centre for Atherothrombotic & Metabolic Disease, Hull York Medical School, Hull, UK

## Abstract

The dystrophin-glycoprotein complex (DGC) links the muscle cytoskeleton to the extracellular matrix and is responsible for force transduction and protects the muscle fibres from contraction induced damage. Mutations in components of the DGC are responsible for muscular dystrophies and congenital myopathies. Expression of DGC components have been shown to be altered in many myopathies. In contrast we have very little evidence of whether adaptive changes in muscle impact on DGC expression. In this study we investigated connection between muscle fibre phenotype and the DGC. Our study reveals that the levels of DGC proteins at the sarcolemma differ in highly glycolytic muscle compared to wild-type and that these changes can be normalised by the super-imposition of an oxidative metabolic programme. Importantly we show that the metabolic properties of the muscle do not impact on the total amount of DGC components at the protein level. Our work shows that the metabolic property of a muscle fibre is a key factor in regulating the expression of DGC proteins at the sarcolemma.

## Introduction

Skeletal muscle has an elaborate network of proteins responsible for translating the shortening of the sarcomeres into force capable of moving bones to permit locomotion. Furthermore it protects the muscle fibre from damage during contraction^1^. These proteins, collectively known as the dystrophin-glycoprotein complex (DGC), link the cytoskeleton to laminin in the extracellular matrix (ECM)^1,2^. Dystrophin, the first identified member of this complex, is a large intracellular protein composed of four functional units (i.e. N-terminus acting binding segment, spectrin repeats, cysteine rich region and the C-terminal portion). Its cysteine rich region binds the transmembrane protein β-Dystroglycan (β-DG). α-DG, an extracellular protein, binds β-DG on the surface of the myofibre. α-DG directly binds a number of ECM component including Laminin and collagen IV in the basal lamina as well as being involved in indirect interactions with interstitial matrix proteins including collagen I (reviewed in^3^). The DGC also contains the members of the Sarcoglycan (SG) family of transmembrane proteins and other components (reviewed in^4^). Mutations in almost any of the DGC genes result in muscle disease e.g. mutations in dystrophin lead to Duchenne and Becker Muscular Dystrophy (DMD/BMD)^5^, mutations in DG and SGs lead to a variety of Limb-Girdle Muscular Dystrophies and other pathologies^6,7^.

The relationship between muscle and the ECM in a disease context is of upmost importance as it has clinical implications. Fibrosis is a key feature of DMD and other myopathies^3,8^. Furthermore a longitudinal study of DMD patients concluded that endomysial fibrosis was the only myopathologic parameter that significantly correlated with poor motor outcome^9^ and numerous strategies have been developed to control this feature in order to improve clinical features of this disease^10^. However, the treatments based on controlling fibrosis in neuromuscular diseases must also consider the normal function of connective tissue in maintaining muscle fibre function and survival. It is well known that there is a correlation between fibre type and propensity to undergo necrosis in DMD; with fast fibres being preferentially affected in DMD^11^. As fibres in DMD are thought to be damaged by contraction mediated damage^12^ and that the fast fibres these are the ones with the least amount of endomysial ECM it is worthy of contemplation where increasing the support nature of the endomysium without evoking a fibrotic reaction may confer fibre robustness.

Skeletal muscle is an adaptable tissue which changes in terms of composition and mass in reaction to mechanical, electrical and chemical stimuli^13^. It is important to comprehend that muscle responds to stimuli by modifying both its contractile elements (muscle fibres) and invariably all other tissue components including the blood supply and ECM. This is exemplified by the phenotype of mice lacking the TGF-ß family protein Myostatin (Mstn), a member of the TGF-ß family of secreted proteins. Myostatin is a potent inhibitor of muscle development and oxidative metabolism. Loss of function mutation in the mouse *Mstn* gene leads not only to hypertrophic/glycolytic/fast contracting fibres^14,15^ but also to a decrease in blood vessels and significantly lower levels of ECM components compared to wild-type (WT)^16^. Changes in the ECM of *Mstn*^−/−^ mice could be attributed to either (1) muscle fibre enlargement not being accompanied by a commensurate rise in ECM synthesis, or (2) an adaptive change to harmonise ECM to the physiology of muscle. In support of the latter, numerous investigations have suggested that fast/glycolytic contracting muscle fibres that tend to be larger in cross sectional area are surrounded by less ECM than slow/oxidative contracting fibres^17,18^.

Several studies have shown that the DGC and ECM undergo profound changes in organisation in a disease context^19,20^. Yet few have examined the impact of physiological adaptation of muscle on the force transduction apparatus. Here we used two innovations to address the dearth of knowledge in this field. The first is an allelic series that differ in their metabolic properties of muscle fibres^21^. The series was made by using the *Mstn*^−/−^ background as well as the progeny after crossing it with a line expressing Estrogen-related receptor gamma (Errγ), a master regulator of oxidative metabolism. Errγ is a transcription factor expressed at elevated levels in slow muscle and in tissues with high oxidative metabolism levels. Here it supports mitochondrial activity and biogenesis^22,23^. We have shown that over-expression of Errγ on the myostatin null background results in hypertrophic fibres that express MHCIIB and yet robustly express Succinate Dehydrogenase (SDH), a marker of oxidative metabolism. This contrasts with the hypertrophic fibres of the myostatin null mouse which tend to express MHCIIB and are glycolytic. These two models facilitate comparison of morphological characteristics (i.e. the DGC and ECM) between fibres with the same contraction profile (i.e. MHC composition) yet which differ in their metabolic status (i.e. oxidative versus glycolytic). Second is the application of semi-quantitative immunofluorescence methods that allow the investigator to define the amounts of sarcolemma components and relate this to fibre phenotype^24,25^. The present investigations reveals that fibre type affects the levels of DGC and ECM components. Secondly, sarcolemmal levels of all DGC proteins investigated were higher in WT muscle compared to that from the hypertrophic *Mstn*^−/−^. Lastly we provide evidence that the amount of DGC at the sarcolemma were elevated in *Mstn*^−/−^ fibres following the overexpression of Estrogen Related Receptor Gamma (Erry) (*Mstn*^−/−^/*Errγ*^T*g*/+^) which imparts a programme of oxidative metabolism. The results presented show that the metabolic programme of muscle is a factor in the formation of the DGC and ECM. Finally, we show that the amount of protein found at the fibre membrane is not related to the total amount present in the fibre. These results imply that mechanisms that facilitate protein shuttling are important in the development of the force transduction apparatus.

## Results

### Quantification of inter-fibre collagen in wild-type mouse skeletal muscle

**Figure 1:**
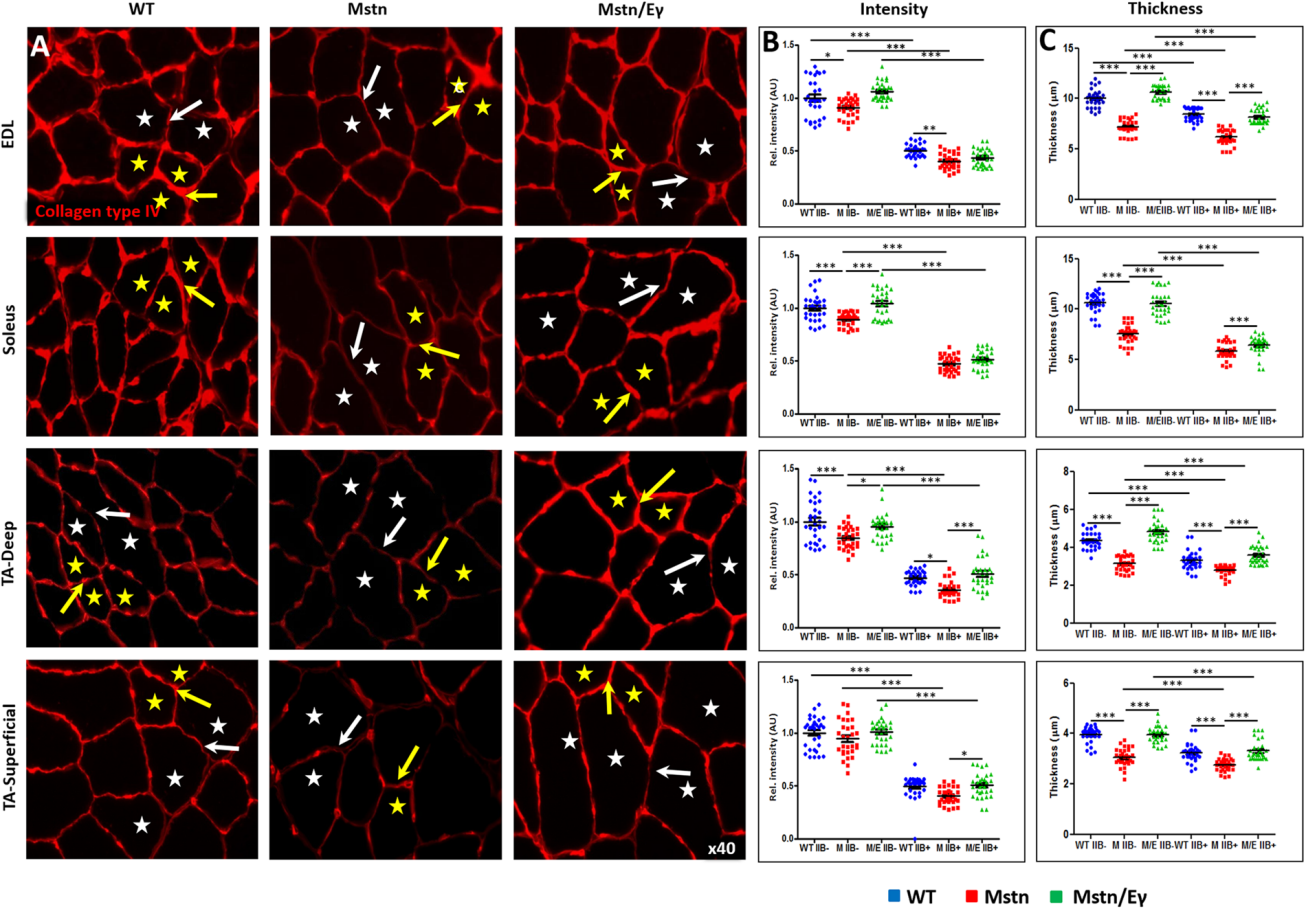
Collagen IV profiles in WT, *Mstn*^−/−^ and *Mstn*^−/−^/*Errγ*^T*g*/+^ mouse muscle. (**A**) Expression of collagen IV in relation to MHC fibre type. Serial sections processed with an anti-MHCIIB antibody to identify MHCIIB^−^ and MHCIIB^+^ fibres (see Supplementary Figures). Representative MHCIIB^−^ indicated with yellow stars and MHCIIB^+^ fibres by white stars. Expression of collagen IV was more robust in ECM between two MHCIIB^−^ fibres (yellow arrow) compared to that between MHCBII^+^ fibres (white arrows) in wild type muscle. The same relationship albeit at lower levels was noted in *Mstn*^−/−^ muscle. Expression levels were increased in *Mstn*^−/−^ by *Errγ* in ECM between MHCIIB^−^ (yellow arrows) as well as MHCIIB^+^ (white arrows) compared to *Mstn*^−/−^ fibres. (**B**) Intensity of collagen IV expression quantified by setting standard value of 1 for the level between MHCIIB^−^ fibres from WT mice. (**C**) Collagen IV expression also quantified in terms of thick domain. n = 30 from each cohort. *p < 0.05, **p < 0.01, and ***p < 0.001. Statistical analysis performed by one-way ANOVA followed by Bonferroni correction of multiple comparisons.

By means of cryo-scanning electron microscopy, our previous work demonstrated that the endomysium of the *Mstn*^−/−^ mouse is thinner in comparison to sex and age- matched wild-type mice (WT)^16^. Additionally we have shown that collagen I and IV are deposited at higher levels between MHCIIB^−^ relative to between MHCIIB^+^ fibres, a relationship that held true in both WT and *Mstn*^−/−^ muscles^16^. Here, we used florescence microscopy-based techniques to determine how they compared to more laborious electron microscopic approaches to quantify the ECM content of skeletal muscle. Using these techniques, we firstly assessed the levels of collagen IV as well as the endomysium thickness domain in the between MHCIIB^−^ fibres relative to MHCIIB^+^ fibres of WT mice. (Note: It was not possible perform double immunofluorescence for MHCIIB and collagen IV due to the availability of resolving secondary antibodies. Therefore, serial sections were individually stained for the two markers and MHCIIB^+^ fibres marked on the collagen profile (also the case for laminin profiling). Individual staining of MHCIIB, MHCIIA and MHCI are shown in Supplementary Figs 1–3. For DGC components, composite images are presented with simultaneous staining of sarcolemma protein and MHCIIB expression.) Measurements were made from three muscles with differing metabolic profiles as well as MHC distribution (Extensor Digitorum Longus (EDL), soleus, deep and superficial portions of the tibialis anterior (TA) muscle). In these muscles the contraction speed based on MHC expression, going from fastest to slowest, observes the following order: superficial TA, EDL, deep TA, soleus. In each case investigations were made in four non-overlapping regions. Measurement of both fluorescence signal intensity and thickness in wild type muscles indicated that there was more collagen IV between MHCIIB^−^ fibres compared to MHCIIB^+^ fibres (Fig. 1A–C, note that WT soleus did not contain any MHCIIB^+^ fibres), a finding that is in agreement with our previous findings using electron microscopy^16^.

### Impact of *Mstn*^−/−^/*Errγ*^T*g*/+^ on inter-fibre collagen

We next analysed *Mstn*^−/−^ muscles by fluorescence microscopy. Immunolabeling revealed an general reduction in collagen IV around the fibres. Like our findings with cryo-electron microscopy, immunofluorescence staining corroborated that the variance in collagen IV levels between MHCBII^−^ and MCHBII^+^ fibres is not influenced by the genetic deletion of *myostatin* (Fig. 1A–C). These results indicate that the deletion of myostatin results in an overall decrease in collagen IV levels that similarly affects MHCBII^+^ and MCHBII^−^ fibres.

Given the excellent correlation between electron microscopy and fluorescence microscopy quantifications, we used immunofluorescence to assess the impact of the super-imposition of an oxidative programme on collagen IV deposition in the *Mstn* null background (*Mstn*^−/−^/*Errγ*^T*g*/+^). We found that forced expression of *Errγ* normalised collagen IV levels in *Mstn*^−/−^ mice levels that were indistinguishable from WT levels, irrespective of MHCIIB expression (Fig. 1A–C). Of note, *Errγ* over-expression on the *Mstn* null background did not correct the aberrant presence of MHCBII^+^ fibres in the soleus muscle. Expression of an interstitial component of the ECM, collagen I, showed the same pattern as collagen IV (a basal lamina protein) with regards to MHCIIB expression and genotype (Supplementary Fig. 1).

### Relationship between DGC and laminin and *Mstn*^−/−^/*Errγ*^T*g*/+^ on skeletal muscle

We next examined intracellular, membrane-associated and extracellular components of the DGC that link the myofibre cytoskeleton to the collagens with a view of establishing a relationship between expression levels, fibre size and metabolic activity mediated in the *Mstn*^−/−^/*Errγ*^T*g*/+^. To that end we profiled the expression of dystrophin as an intracellular component, β-dystroglycan (βDG), four sarcoglycans (SG, α, β, γ and δ) as representatives of membrane associated polypeptides as well as α-dystroglycan (αDG) and laminin as extra-cellular entities in the EDL, soleus and TA muscles of the three cohorts.

**Figure 2:**
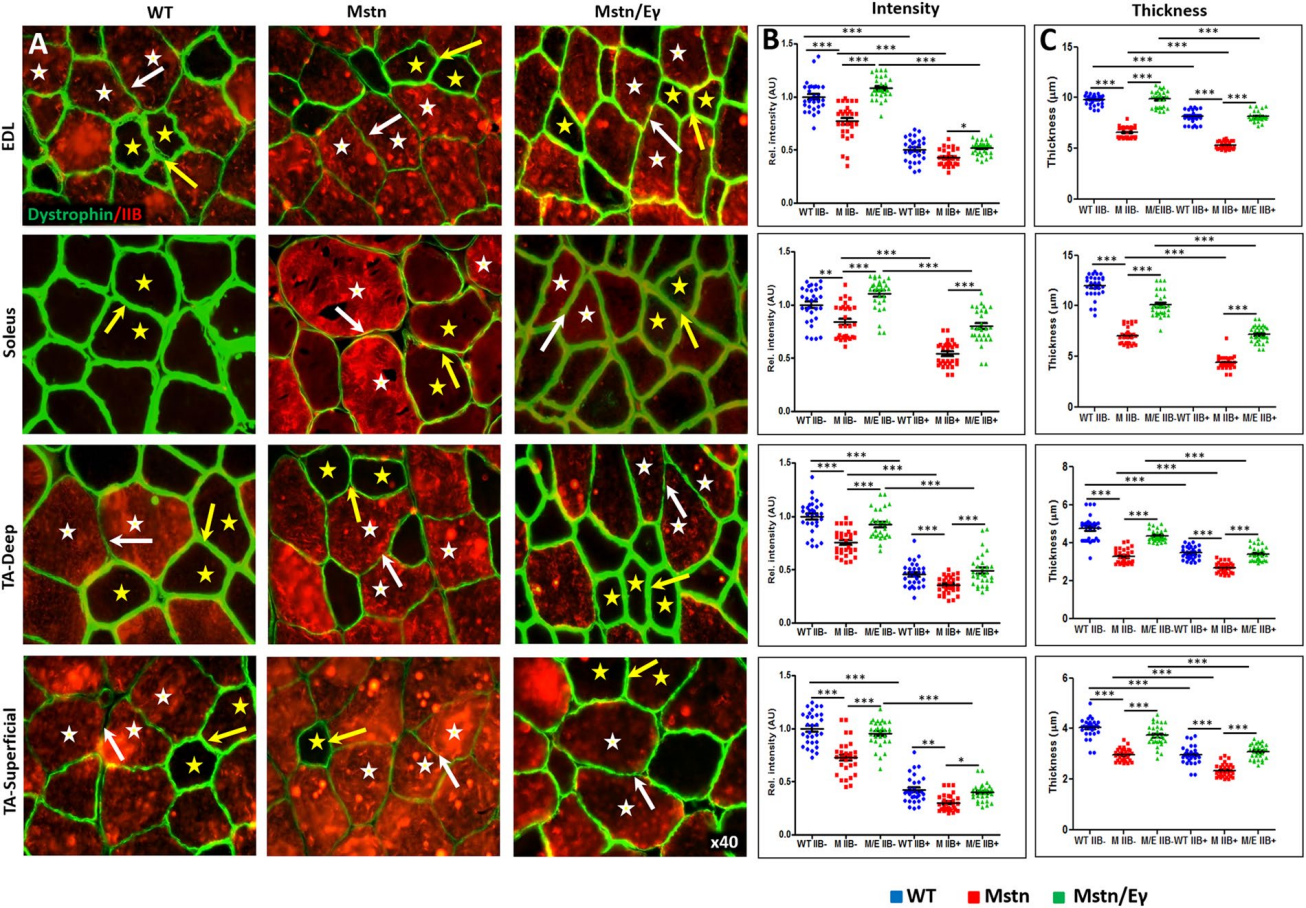
Dystrophin profiles in WT, *Mstn*^−/−^ and *Mstn*^−/−^/*Errγ*^T*g*/+^ mouse muscle. (**A**) Immunofluorescence profile of Dystrophin in relation to MHC fibre type. MHCIIB expression in red. Representative MHCIIB^−^ fibres are indicated by yellow stars and MHCIIB^+^ fibres by white stars. Expression of Dystrophin was stronger in ECM between two MHCIIB^−^ fibres (yellow arrow) compared to between MHCBII^+^ fibres (white arrows) in wild type muscle. Same relationship albeit at lower levels in *Mstn*^−/−^ expression between MHCIIB^−^ fibres and MHCIIB^+^ fibres. Expression increased in *Mstn*^−/−^ by *Errγ* in ECM between MHCIIB^−^ (yellow arrows) as well as between MHCIIB^+^ (white arrows) fibres compared to those from *Mstn*^−/−^ mice. (**B**) Expression of Dystrophin quantified by intensity by setting standard value of 1 for the level between MHCIIB^−^ fibres from WT mice. (**C**) Dystrophin expression quantification in terms of thickness. n = 30 for each cohort. *p < 0.05, **p < 0.01, and ***p < 0.001. Statistical analysis performed by one-way ANOVA followed by Bonferroni correction of multiple comparisons.

**Figure 3:**
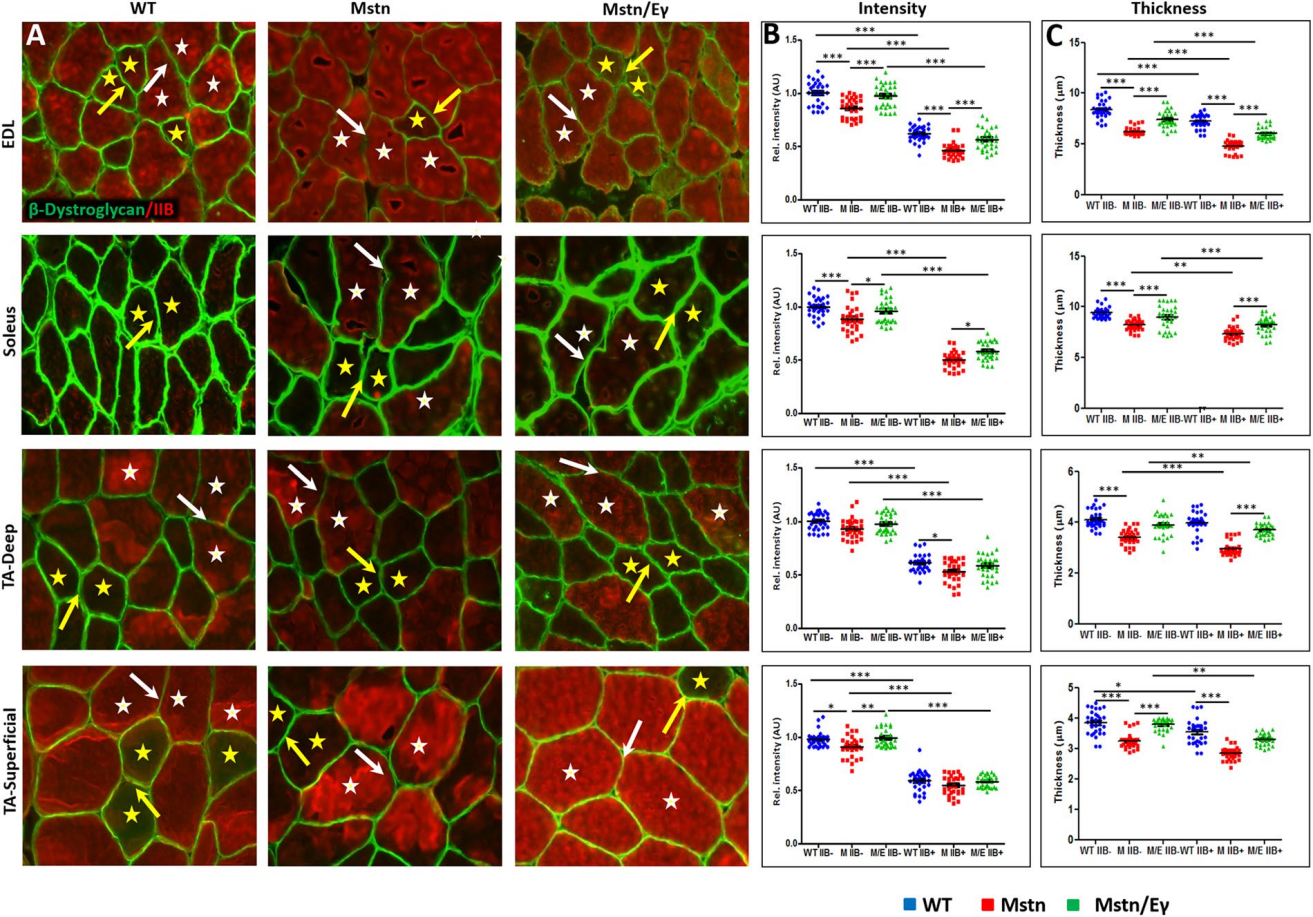
ß-Dystroglycan profiles in WT, *Mstn*^−/−^ and *Mstn*^−/−^/*Errγ*^T*g*/+^ mouse muscle. (**A**) Immunofluorescence profile of ß-Dystroglycan in relation to MHC fibre type. MHCIIB expression in red. Representative MHCIIB^−^ indicated by yellow stars and MHCIIB^+^ fibres by white stars. Note higher of ß-Dystroglycan levels in all genotypes between MHCIIB^−^ (yellow arrows) compared to MHCIIB^+^ (white arrows). (**B**) Expression of ß-Dystroglycan quantified by intensity by setting standard value of 1 for the level between MHCIIB^−^ fibres from WT mice. (**C**) ß-Dystroglycan expression quantification in terms of thickness domain. n = 30 from each cohort. *p < 0.05, **p < 0.01, and ***p < 0.001. Statistical analysis performed by one-way ANOVA followed by Bonferroni correction of multiple comparisons.

**Figure 4:**
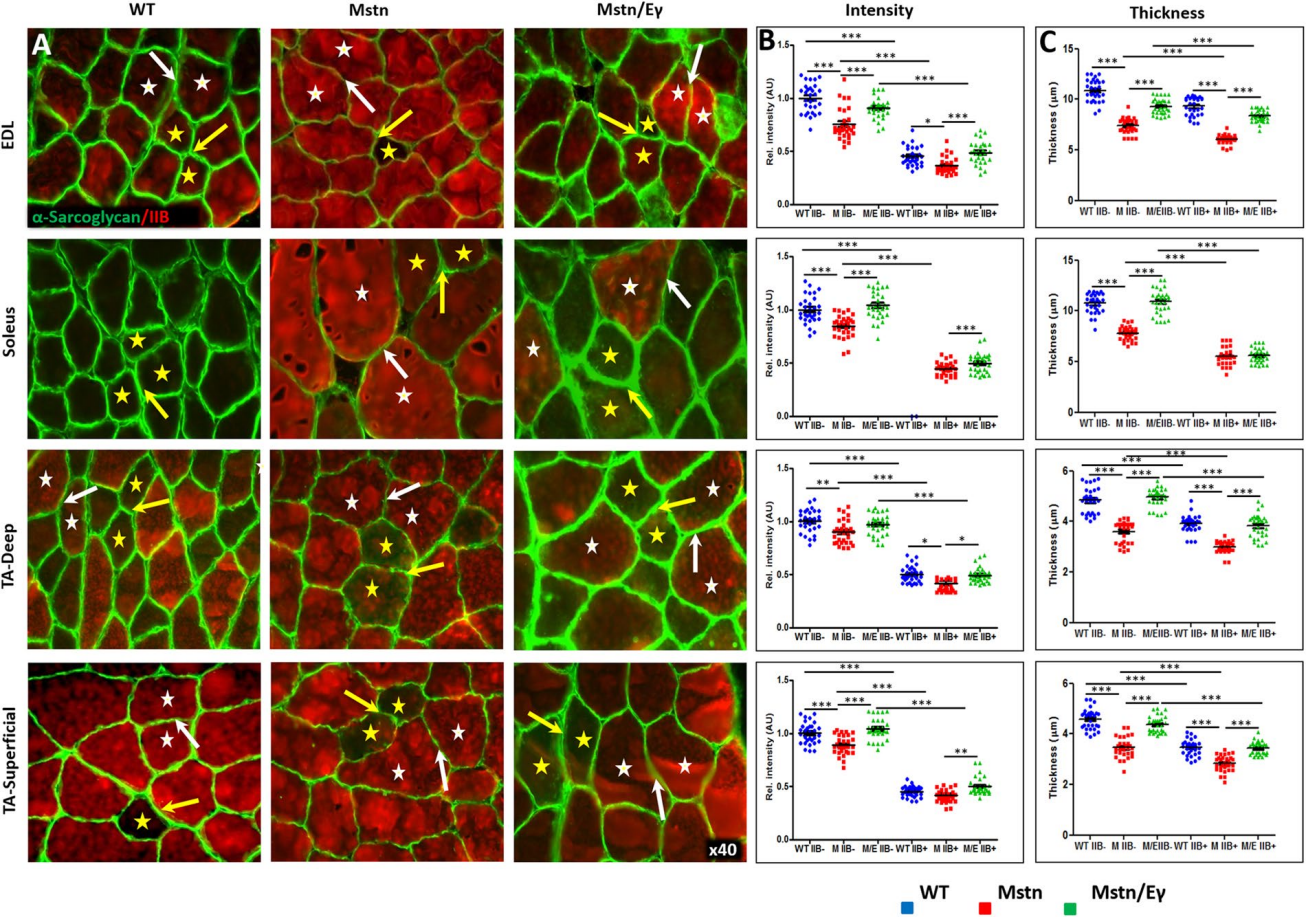
α-Sarcoglycan profiles in WT, *Mstn*^−/−^ and *Mstn*^−/−^/*Errγ*^T*g*/+^ mouse muscle. (**A**) Immunofluorescence profile of α-Sarcoglycan in relation to MHC fibre type. MHCIIB expression shown in red. Representative MHCIIB^−^ fibres indicated by yellow stars and MHCIIB^+^ fibres by white stars. Note higher levels of α-Sarcoglycan in all genotypes between MHCIIB^−^ (yellow arrows) compared to MHCIIB^+^ (white arrows). (**B**) Expression of α-Sarcoglycan quantified by intensity by setting standard value of 1 for the level between MHCIIB^−^ fibres from WT mice. (**C**) α-Sarcoglycan expression quantification in terms of thick domain. n = 30 from each cohort. *p < 0.05, **p < 0.01, and ***p < 0.001. Statistical analysis performed by one-way ANOVA followed by Bonferroni correction of multiple comparisons.

**Figure 5:**
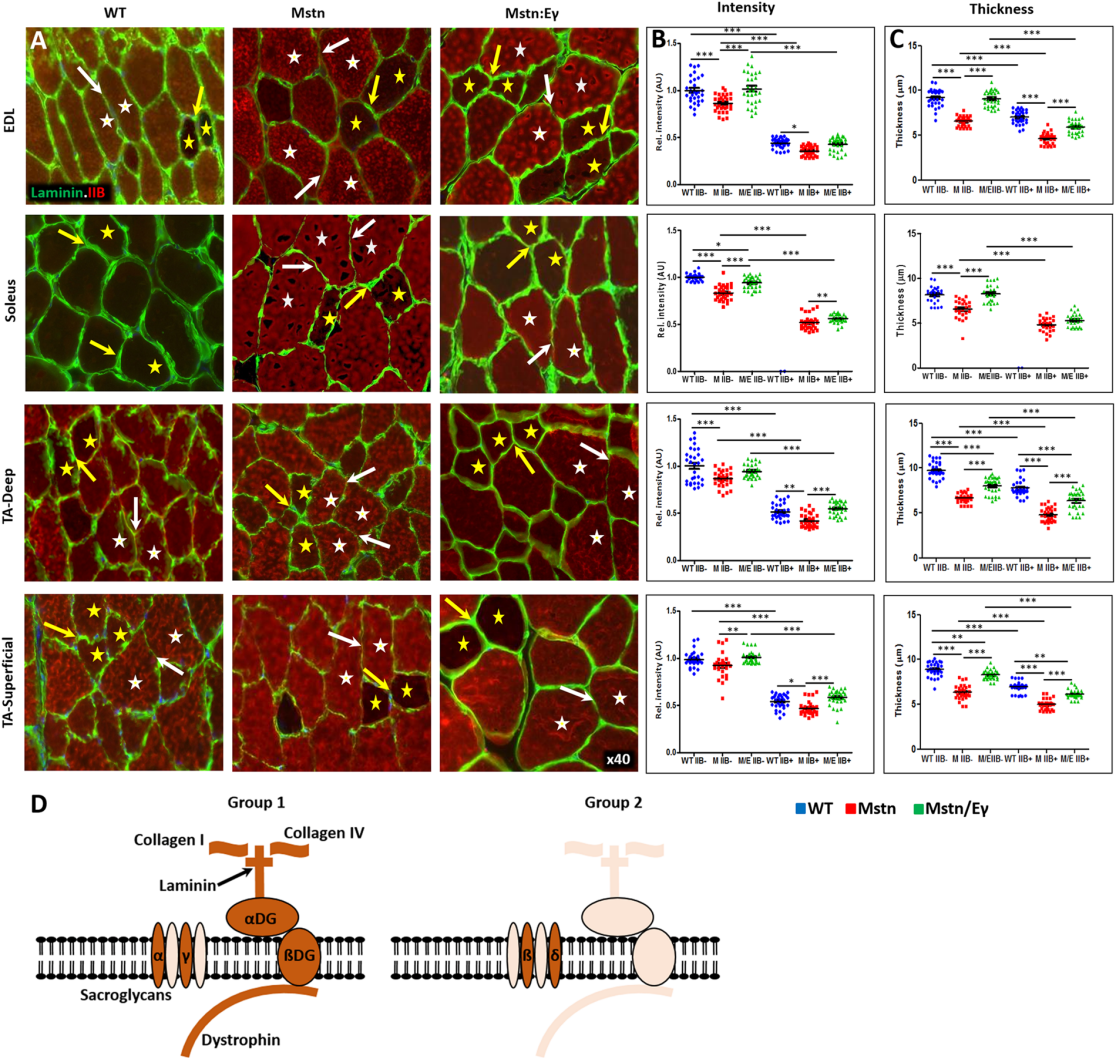
Laminin profiles in WT*, Mstn*^−/−^ and *Mstn*^−*/*−^/*Errγ*^T*g*/+^ mouse muscle. (**A**) Immunofluorescence profile of laminin in relation to MHC fibre type. MHCIIB expression in red. Representative MHCIIB^−^ fibres indicated by yellow stars and MHCIIB+ fibres by white stars Note higher levels of Laminin in all genotypes between MHCIIB^−^ (yellow arrows) compared to MHCIIB^+^ (white arrows). (**B**) Expression of Laminin quantified by intensity by setting standard value of 1 for the level between MHCIIB^−^ fibres from WT mice. (**C**) Laminin expression quantification in terms of thick domain. (**D**) Graphic presentation of DGC and abundance revealed in this study. Dark shading represents increased levels detected at sarcolemma through immunofluorescence compared to lighter shading. n = 30 from each cohort. *p < 0.05, **p < 0.01, and ***p < 0.001. Statistical analysis performed by one-way ANOVA followed by Bonferroni correction of multiple comparisons.

Our study revealed that amount of these proteins in relation to MHCIIB expression and genotype could be categorised into two groups. Group 1 consisted of proteins where the levels in relation to MHCIIB expression and genotype were like to collagens I and IV. This group consisted of dystrophin (Fig. 2), α and βDG (Supplementary Fig. 5 and Fig. 3), α and γSG (Fig. 4 and Supplementary Fig. 6) as well as laminin (Fig. 5). Expression of Group 1 proteins in MHCIIB^−^ fibres was greater, (assessed by intensity measures and thickness) compared to MHCIIB^+^ fibres in WT muscle (panels B and C in Figs 2–5, 5D and Supplementary Figs 5 and 6). Secondly, Group 1 protein expression was lower in *Mstn*^−/−^ fibres when compared to their wild-type MHCIIB^−^/MHCBII^+^ counterparts (panels B and C in Figs 2–5 and Supplementary Figs 5 and 6). Finally, super-imposing oxidative metabolism through *Errγ* expression on the *Mstn*^−/−^ background resulted in major change in Group 1 proteins levels. *Mstn*^−/−^/*Errγ*^T*g*/+^ MHCIIB^−^ and *Mstn*^−/−^/*Errγ*^T*g*/+^ MHCIIB^+^ fibres showed higher levels of expression than their *Mstn*^−/−^ counterparts. Furthermore in many cases these reached levels displayed by WT fibres (panels B and C in Figs 2–5 and Supplementary Figs 2, 5 and 6).

**Figure 6:**
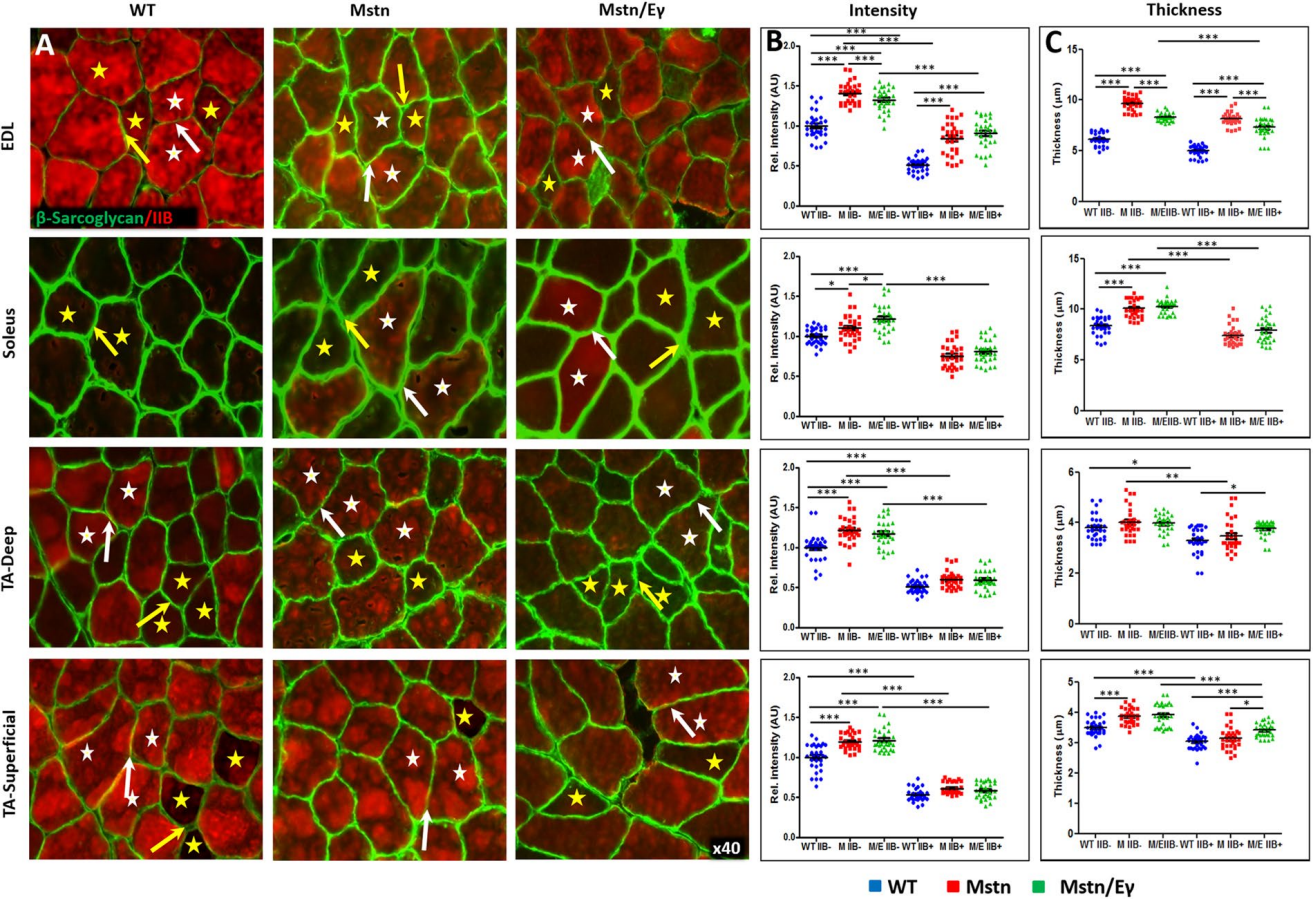
ß-Sarcoglycan profiles in WT*, Mstn*^−*/*−^ and *Mstn*^−/−^/*Errγ*^T*g*/+^ mouse muscle. (**A**) Immunofluorescence profile of ß-Sarcoglycan in relation to MHC fibre type. MHCIIB expression identified by bright red colouring. Representative MHCIIB^−^ fibres indicated by yellow stars and MHCIIB^+^ fibres by white stars. Note higher levels of ß-Sarcoglycan in all genotypes between MHCIIB^−^ (yellow arrows) compared to MHCIIB^+^ (white arrows). (**B**) Expression of ß-Sarcoglycan quantified by intensity by setting standard value of 1 for the level between MHCIIB^−^ fibres from WT mice. (**C**) ß-Sarcoglycan expression quantification in terms of thick domain. n = 30 from each cohort. *p < 0.05, **p < 0.01, and ***p < 0.001. Statistical analysis performed by one-way ANOVA followed by Bonferroni correction of multiple comparisons.

However, the expression of β and δSG failed to follow the trend of Group 1 proteins. Therefore, these were assigned to Group 2 molecules. Group 2 proteins like Group 1, Group 2 polypeptides displayed greater expression in MHCIIB^−^ fibres in comparison to MHCIIB^+^ fibres (panels B and C in Fig. 6, Supplementary Fig. 7 and graphically presented in Fig. 5D). Nevertheless, the expression of Group 2 proteins was greater in *Mstn*^−/−^ fibres in comparison to their MHCIIB^−^/MHCBII^+^ counterparts derived from WT muscles (panels B and C in Fig. 6 and Supplementary Fig. 7). Lastly our study showed that the Group 2 protein expression in *Mstn*^−/−^ fibres was largely unaffected by the expression of *Errγ* (panels B and C in Fig. 6 and Supplementary Fig. 4).

### Impact of *Mstn*^−/−^/*Errγ*^T*g*/+^ on total transcript level and muscle protein for key components of the DGC

**Figure 7:**
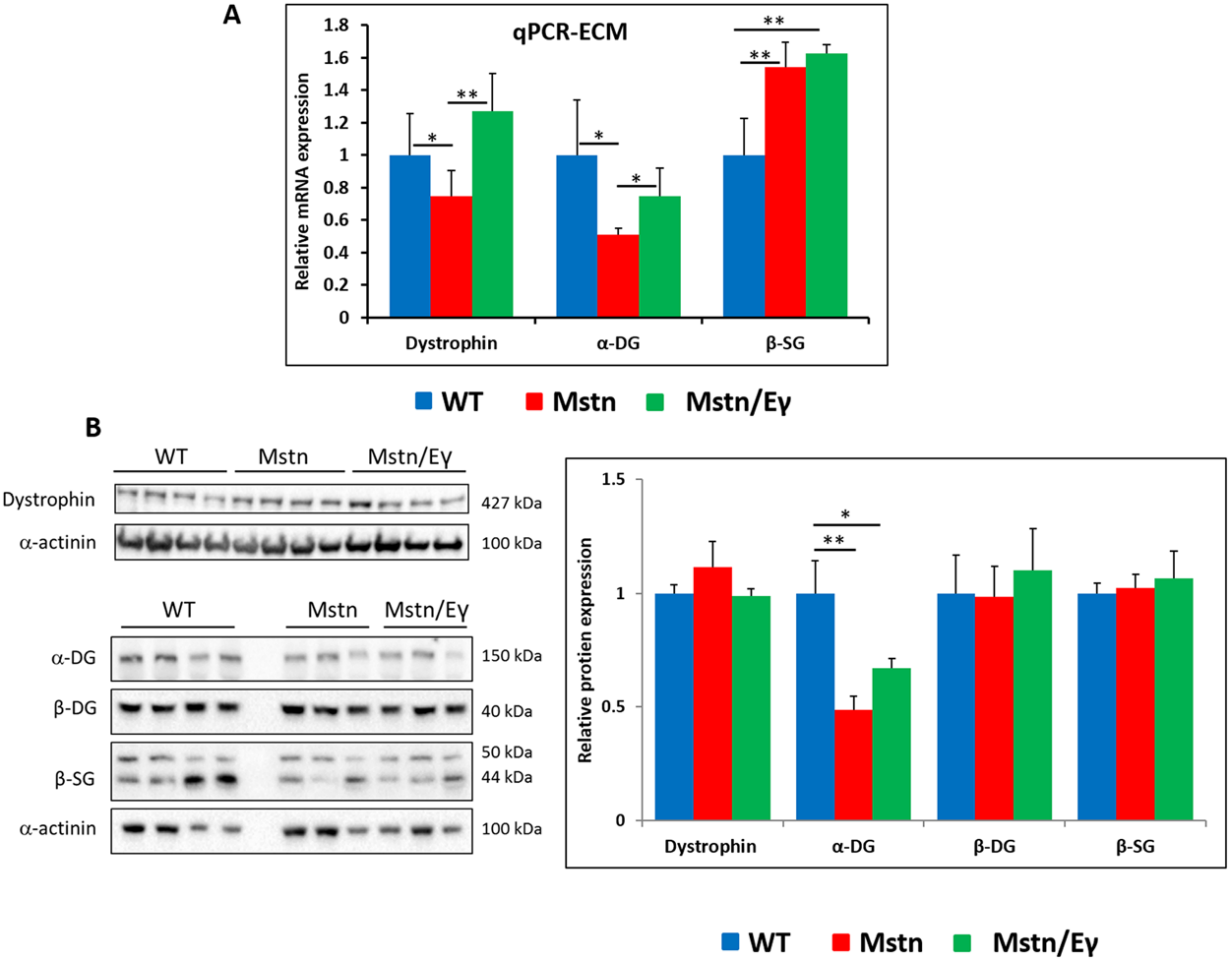
Molecular and Western blot profiling of Soleus ECM associated molecules. (**A**) qPCR profile of DPC associated genes in soleus muscles n = 4 muscle. (**B**) Western blot analysis of protein extracts from soleus muscles from 4 wild type, 6 Mstn^−/−^ and 6 Mstn^−/−^/Errγ^T*g*/+^ mice. Membranes were probed for dystrophin and α-actinin, or cut into strips to allow simultaneous probing of α-DG/β-DG/β-SG/α-actinin. Membrane images were cropped for clarity, but full membranes are shown in Supplementary Fig. 8. Densitometric quantification of protein bands was performed on non-saturated images. Values were normalised to α-actinin and then expressed as a ratio to WT samples run on the same gel (see materials and methods for details). For statistical comparisons, a one-way ANOVA followed by pair-wise comparisons using the Bonferroni correction was performed. *p < 0.05, **p < 0.01, and ***p < 0.001.

Having shown that levels of components of the DGC present at the sarcolemma vary in the three mouse strains, we examined whether this reflected the amount of transcript or the total amount of a specific protein in muscle. To that end we examined their levels in the soleus muscle as it displayed strain dependent variations at the sarcolemma for most of the molecules. We found that at the transcript level, there was good correlation between amounts of RNA molecules for Dystrophin, DG and βSG to their abundance at the sarcolemma in the soleus muscle from the three stains e.g. levels of dystrophin transcript and the protein at the sarcolemma were higher in WT and *Mstn*^−/−^/*Errγ*^T*g*/+^ compared to *Mstn*^−/−^ (Fig. 7). Next, we investigated the total amount of a subset of DGC proteins in the soleus to determine whether this reflected the amount of transcript and its abundance at the sarcolemma. We found that there was no correlation between total amount of Dystrophin, βDG or βSG and their abundance at the sarcolemma (Fig. 7). The level of expression of these three proteins was similar among all three strains. By contrast, levels of αDG were significantly decreased in *Mstn*^−/−^ and *Mstn*^−/−^/*Errγ*^T*g*/+^ samples compared to wild type, in agreement with transcript and immunohistochemical finding. Interestingly, αDG levels were assayed using the IIH6 antibody that only recognises the mature, fully glycosylated form of αDG, that is normally present at the membrane. Therefore, these results indicate that the total amount of DGC proteins in muscle as detected by Western blot analysis does not vary with muscle metabolism. However, their transcription and localisation to the sarcolemma are affected.

## Discussion

Our results firstly show that fibre type, specifically MHCIIB expression, effects levels of sarcolemmal DGC proteins. Secondly, we show that the Mstn phenotype alters the abundance of DGC proteins at the sarcolemma. Most importantly we showed that initiation of a programme that drives oxidative phosphorylation in muscle was able to reverse the changes of membrane levels for many DGC components in *Mstn*^−/−^ muscle. We show that DGC and ECM components can be divided into two groups. Group I (collagen I, IV, dystrophin, α and βDG, α and γSG, laminin) proteins were present at higher at the sarcolemma in wild-type fibres than those from *Mstn*^−/−^ mice for a specific MHCII isoform. However, in Group 2 (ß and δSG) this relationship is reversed.

The development of easy to use techniques have now enabled investigators to examine the association between metabolism and the ECM and metabolism at single fibre resolution. In this study we deployed immunofluorescence based approaches to investigate levels of major components of the force transduction apparatus in specific muscle fibre types in a semi-quantitative manner^24,26^. Results from this approach mirror differences in ECM composition in WT and *Mstn*^−/−^ muscles that we previously reported using a more laborious electron microscopy approach^16^. Interestingly, while qPCR detected similar differences at the transcriptional level, findings from immuno-blotting of muscle proteins did not concord our electron microscopy and semi-quantitative immunofluorescence measurements. It is important to recognise that microscopy studies concentrated on components specifically at the plasma membrane. In contrast Western blot analyses were performed on total muscle lysates. The lack of correlation between the protein levels at the sarcolemma and total protein content suggests that the mechanism responsible for trafficking plays a major role in the assembly of the DGC. Furthermore, these results may have relevance in assessing the efficacy of therapies for diseases related to DGC proteins. Our results suggest that total muscle protein content may not be a good metric for efficacy as it is possible to have high overall levels of DGC components without them localising to the sarcolemma. Indeed many studies now use proper localisation of DGC to the sarcolemma as a measure of therapy efficacy^27^. Furthermore, it highlights that a better understanding of the mechanism responsible for DGC translocation to the sarcolemma could be exploited to increase the efficacy of dystrophin restoration-based therapies.

Our previous work has shown that muscle from *Mstn*^−/−^ mice contained less collagen than WT. Those findings could be explained either by the constraints placed on a cell through the limited functional capacity of the nucleus (e.g. the karyoplasmatic hypothesis of Strassburger and Hertwig amongst other^28,29^) or as a consequence of the development of muscle with rapid contraction characteristics underpinned by glycolytic metabolism following gene deletion^30^. In the former, the muscle fibre would undergo enlargement without a commensurate increase in the amount of collagen. In the latter, fast contracting muscle fibres should always be bestowed with lower levels of ECM so to decrease the proportion of energy stored in the connective tissue, making more available for work^30^. Indeed, it has been shown by a number of investigators that fast muscle has less ECM than slow^17,31^. However, in our previous study we were unable to define the influence of fibre type on collagen levels and it was limited to only extracellular components of the force transduction apparatus. Here we show that unrelatedly to genotype, MHCIIB^−^ fibres have greater amounts of DGC than MHCIIB^+^ fibres. Secondly, we show that inducing oxidative phosphorylation in hypertrophic fibres alters the expression of sarcolemmal DGC proteins. It is critical to keep in mind we are detecting effects that are not linked to fibre type switching. These findings highlight an original and unforeseen result. According to the work of Kovanen and colleagues^17,30,31^, one would predict that all MHCII myofibres, irrespective of genotypes, should contain similar levels of DGC proteins. We show that this prediction is violated. Our work points to the fact that metabolic programme of muscle has primacy over MHCII expression with regards to DGC expression.

The examination of both the intercellular and extracellular components of the DGC and the ECM reveals a possible influence of the muscle metabolic programme on both the muscle fibre and their fibroblasts, major producers of collagen^32–34^. The important feature here is that metabolic programme must harmonises the production of DGC and ECM from these two cellular sources in keeping with the muscle (oxidative/glycolytic) phenotype. This study reveals a fascinating relationship between muscle and fibroblasts. Previous work has shown that myostatin is an inhibitor of muscle hypertrophy^14^ but promotes muscle fibroblast proliferation^35^. Therefore, it would be predicted that in the absence of myostatin, muscle would be hypertrophic and invested with less ECM due to the decreased number of collagen secreting fibroblasts, which is in fact what is observed. However, the remodelling of *Mstn*^−/−^ muscle following *Errγ* expression demonstrates that oxidative metabolism could overcome the effects of the absence of *Mstn* on fibroblasts.

Our understanding of the aetiology of ECM in undamaged muscle is poor compared to myogenic or neurogenic development^2^. ECM components are produced by numerous cells in muscle including myogenic cells^36^, satellite cells^37^ and Schwann cells^38^ but it is believed that it is the fibroblast population that not only produce most of the ECM but also organise it into a functional unit^39^. However, to our knowledge there is no study describing the role of factors that control the formation of ECM and which are produced by myofibres that are dependent on metabolic programmes. In other systems, for example the skin, it is known that overly active oxidative programme result in the remodelling of the ECM through the secretion of metallomatrix proteases^40^. We clearly show that metabolic properties of muscle fibres influence ECM production and suggest that the allelic series used in this study could be a means of investigating this topic. In future we intend to isolate single muscle fibres from mice used in this investigation and using a combination of transcriptome and bio-informatic approaches, identify pathways and (secreted) molecules that originate from myogenic cells that regulate ECM synthesis in fibroblasts

Our work shows that endomysial ECM is regulated by the metabolic programme of the muscle fibre. We propose that increasing the quantity of the DGC and EMC in fibres that are prone to contraction mediated damage (fast fibres) could be of clinical value. Herein we suggest that increasing the oxidative properties of fast fibres in DMD will result in more connective tissue that may translate into greater resilience to contraction mediated damage. We are nevertheless mindful that any intervention should only increase endomysial ECM and not promote fibrosis.

Our study using *Mstn* mutation reveals an interesting feature related to the composition of the DGC. Most DGC models are depicted as having equimolar contribution of each component to this complex (e.g.^41^). In line with our previous report of multiple and independent dystroglycan and dystrophin complexes^42^, we demonstrate here that there can be variability in DGC complex at the sarcolemma. Here, we detected increased levels of βSG and δSG in *Mstn*^−/−^ fibres compared to other DGC components (Fig. 6, Supplementary Fig. 7 and graphic in Fig. 5). The biological relevance of changes DGC complex stoichiometry in *Mstn*^−/−^ mutant have yet to be established. It is undoubtedly significant as many studies have shown that the sarcoglycan complex protects the muscle fibre from damage^43^. As each sarcoglycan has a unique property we suggest that there is a functional significance for altering the sarcoglycan content in hypertrophic fibres based on the findings showing that mutations in each sarcoglycan manifests in a specific disease (limb-girdle muscular dystrophy type (LGMD) 2D, 2E, 2C and 2F are caused by mutations in α, ß, γ, δ respectively^7^).

In summary our work that demonstrates that the DGC is a highly adaptable structure that responds to the metabolic nature of the muscle fibre. In future this approach will be extended to explore the relationship of changes in metabolic activity the muscular dystrophies and sarcopenia and the abundance of DGC and ECM components.

## Methods

### Ethical approval

Experimental procedures were approved by the University of Reading Animal Care and Ethical Review Committee and were conducted under a project license from the United Kingdom Home Office in agreement with the Animals (Scientific Procedures) Act 1986. Animals were humanely sacrificed via Schedule 1 killing between 8:00–13:00.

### Animal maintenance

Healthy C57Bl/6 WT, *Mstn*^−/−^*, Mstn*^−/−^/*Errγ*^T*g*/+^ mice were bred and maintained in accordance to the Animals (Scientific Procedures) Act 1986 (UK) and approved by the University of Reading in the Biological Resource Unit of Reading University. Mice were housed under standard environmental conditions (20–22 °C, 12–12 hr light–dark cycle) and provided food and water ad libitum. We used male mice that were 4–5 months old for this study. Specific experimental group consisted of 4–6 mice.

### Immunohistochemistry

Muscles were dissected following cervical dislocation into PBS. Muscles were snap frozen on a bed of isopentane cooled by liquid nitrogen. Frozen muscles (EDL, Soleus and TA) were mounted for cryo-sectioning in Tissue Tech freezing medium (Jung). 10 µm cryosections were taken using a Bright Cryostat (Bright PLC UK) and placed onto glass microscope slides. Tissue sections were air dried for 30 minutes at room temperature (RT) and either used immediately or stored at −80 °C. Sections were washed three times PBS prior to immunohistochemistry staining. Object slides were treated with permeabilisation buffer (0.952 g Hepes, 0.260 g MgCl_2_, 0.584 g NaCl, 0.1 g Sodium azide, 20.54 g Sucrose and 1 ml Triton X-100 in 200 ml dH_2_O) for 15 minutes at RT, before the application of block wash buffer (PBS with 5% foetal calf serum (v/v), 0.05% Triton X-100) for 30 minutes at RT.

Primary antibodies were diluted in wash buffer and incubated overnight at 4 °C. All secondary antibodies were diluted in wash buffer for minimum of 30 minutes (in dark) prior to their application onto the slides. Sections were then incubated for 1 hr in the dark at room temperature. Finally, slides were mounted in fluorescent mounting medium, and myonuclei were visualised using (2.5 µg/ml) 4,6-diamidino-2-phenylindole (DAPI). List of all antibodies used is provided in the Supplementary Information File.

### Western blot

Fresh muscles were homogenized in lysis buffer (4M Urea, 125 mM Tris pH 6.8, 4% (w/v) SDS, protease inhibitors). Protein content was quantified with the Pierce BCA Protein assay. 20 µg of protein was resolved using 3–8% (for dystrophin) or 4–12% (for all the other DGC proteins) gradient SDS-PAGE mini-gels (pre-made gels from Invitrogen), and thereafter electro-transferred to nitrocellulose membranes (Whatman). Membranes were stained with Ponceau Red S to visualise lanes, check the quality of the protein transfer, and mark the placement of molecular weight markers. For detection of dystrophin, the entire membrane was incubated with a mixture of antibodies against dystrophin and against α-actinin (loading control). For detection of DGC proteins, membranes were cut at appropriate molecular weights to allow simultaneous probing of the same samples with different anti-DGC antibodies, as well as α-actinin. For β-DG and β-SG that have very similar molecular weights, the same membrane was probed sequentially taking advantage of the fact that the two antibodies are raised in different species. The membrane strip was first probed with the rabbit polyclonal antibody to β-SG. After image acquisition, the same membrane strip was incubated overnight at 4 °C in 0.1% (w/v) Tween-20/Tris buffered saline, then probed with the mouse monoclonal antibody to β-DG. α-actinin was used as a loading control because it is expressed only in muscle fibres allowing us to normalise expression of our DAPC proteins to the amount of muscle tissue present in each sample. All membranes were blocked in 5% skim milk in 0.1% (w/v) Tween-20/Tris buffered saline for one hour at room temperature. Membranes were incubated with appropriate primary antibodies overnight at 4 °C followed by three five-minute washes in 0.1% (w/v) Tween-20/Tris buffered saline at room temperature. Thereafter, membranes were incubated at RT with the appropriate horseradish peroxidase-conjugated secondary antibodies (Jackson ImmunoResearch). Protein bands were visualized using enhanced chemiluminescence reagents (BioRad). Signal was detected on a ChemiDoc MP Imaging System (BioRad) at an automated range of exposures ranging from 1–60 seconds. For densitometric analysis, the Image Lab software was used to quantify protein band intensities from non-saturated exposures. Signal intensities for the proteins of interest were normalized to α-actinin probed on the same membrane, on the same exact samples. Final results for *Mstn*^−/−^ and *Mstn*^−/−^/*Errγ*^T*g*/+^ mice were expressed as a ratio to WT samples. Because we could not run all samples on one single gel, the full set of wild type samples (N = 4) were run on all gels to avoid comparisons between samples run on different gels. List of all antibodies used, is given in theSupplementary Information File together with citations of prior publications where the primary antibodies were characterised. Unprocessed images of most of the entire membranes used to generate Fig. 7b are shown in Supplementary Fig. 8 as well as positive and negative controls that were performed to confirm the specificity of the anti-βSG antibody.

### Quantitative PCR

Frozen soleus muscles were homogenised in TRIzol (Fisher) by a tissue homogeniser. RNA was isolated and purified in the RNAeasy Mini Kit (Quigen, Manchester, UK) following the manufacturer instructions. RNA concentrations were measured using the Nanodrop 2000 (Thermo Scientific). Total RNA (5 µg) was reverse-transcribed to cDNA with SuperScript II Reverse Transcriptse (Invitrogen) and analysed by quantitative real-time RT-PCR on a StepOne Plus cycler, using the Applied Biosystems SYBR-Green PCR Master Mix. Primers were designed using the software Primer Express 3.0 (Applied Biosystems). Relative expression was calculated using the ΔΔ*C*_t_ method with normalization to the reference genes *cyclophilin-B* and *hypoxanthine-guanine phosphoribosyltransferase (HPRT)*. All primer sequences are listed in the Supplementary Information File.

### Semi-quantification of Collagen and Dystrophin Glycoprotein Complex (DGC) protein levels by means of immunofluorescence

Signal intensity of protein of interest was measured as previously published^24,25,44^. For this study the membrane signal intensities of approximately 30 muscle fibres of MHC phenotype (IIB^+^ and IIB^−^) in all muscle sections (EDL, Soleus and TA) from WT, *Mstn*^−/−^, *Mstn*^−/−^/*Errγ*^T*g*/+^ mice were measured. 10 random fibres of a specific phenotype from a central region of each muscle (or portion) were counted from three mice. Fiji software was used to measure signal from an area of interest after images have been corrected for background to avoid regions of signal saturation. All images, including internal controls, were taken at the same exposure in line with non-saturatied levels from WT tissue. We ensured that all subsequent images never reached saturation. Relative levels of signal intensity were calculated from individual fibre measurements of the three cohorts normalised to MHCIIB^−^ of WT muscles.

### Sarcolemma thickness measurement

Images taken at the same exposure were used to quantify the expression domain thick for the protein of interest. Expression domain thicknesses between approximately 30 pairs of juxtapositioned fibres of the same MHC phenotypes (IIB^−^/IIB^−^ and IIB^+^/IIB^+^) from EDL, Soleus and TA muscle sections originating from the three genetic cohorts were measured using Fiji software. Data was exported into an Excel spreadsheet.

### Imaging and analysis

Zeiss AxioImager A1 microscope was used to examine immunofluorescent stained sections, and images were captured using an Axiocam digital camera with Zeiss Axiovision computer software version 4.8. Images were transferred to Photoshop for sizing and annotation and exported at TIFF flies.

### Statistical analysis

Data are presented as mean ± SE. Significant differences among groups were analysed by one-way analysis of variance (ANOVA) followed by Bonferroni correction for multiple comparison tests as appropriate. Statistical analysis was performed on GraphPad Prism software. Differences were considered statistically significant at *p < 0.05, **p < 0.01 or ***p < 0.001.

## Supplementary information

Supplementary Information

## Data Availability

All relevant data related to this manuscript are available from the authors upon reasonable request.
