# Supplementary material for: Regulation of the dystrophin-associated glycoprotein complex composition by the metabolic properties of muscle fibres
Source: Sci Rep. 2019 Feb 26;9:2770. doi: 10.1038/s41598-019-39532-4 (PMC6391483; doi:10.1038/s41598-019-39532-4)
Supplement: Supplementary file 1 — Supplementary Information [file 41598_2019_39532_MOESM1_ESM.pdf]

**Supplementary Information**

# Title: Regulation of the dystrophin-associated glycoprotein complex composition by the metabolic properties of muscle fibres

**Authors Saleh Omairi, Kwan-Leong Hau, Henry Collins-Hooper, Charlotte Scott, Sakthivel Vaiyapuri, Silvia Torelli, Federica Montanaro, Antonios Matsakas and Ketan Patel.**

**Supplementary Figure 1.** MHCIIB profiles in WT, *Mstn*$^{-/-}$ and *Mstn*$^{-/-}$/*Errγ*$^{Tg/+}$ mouse muscle.

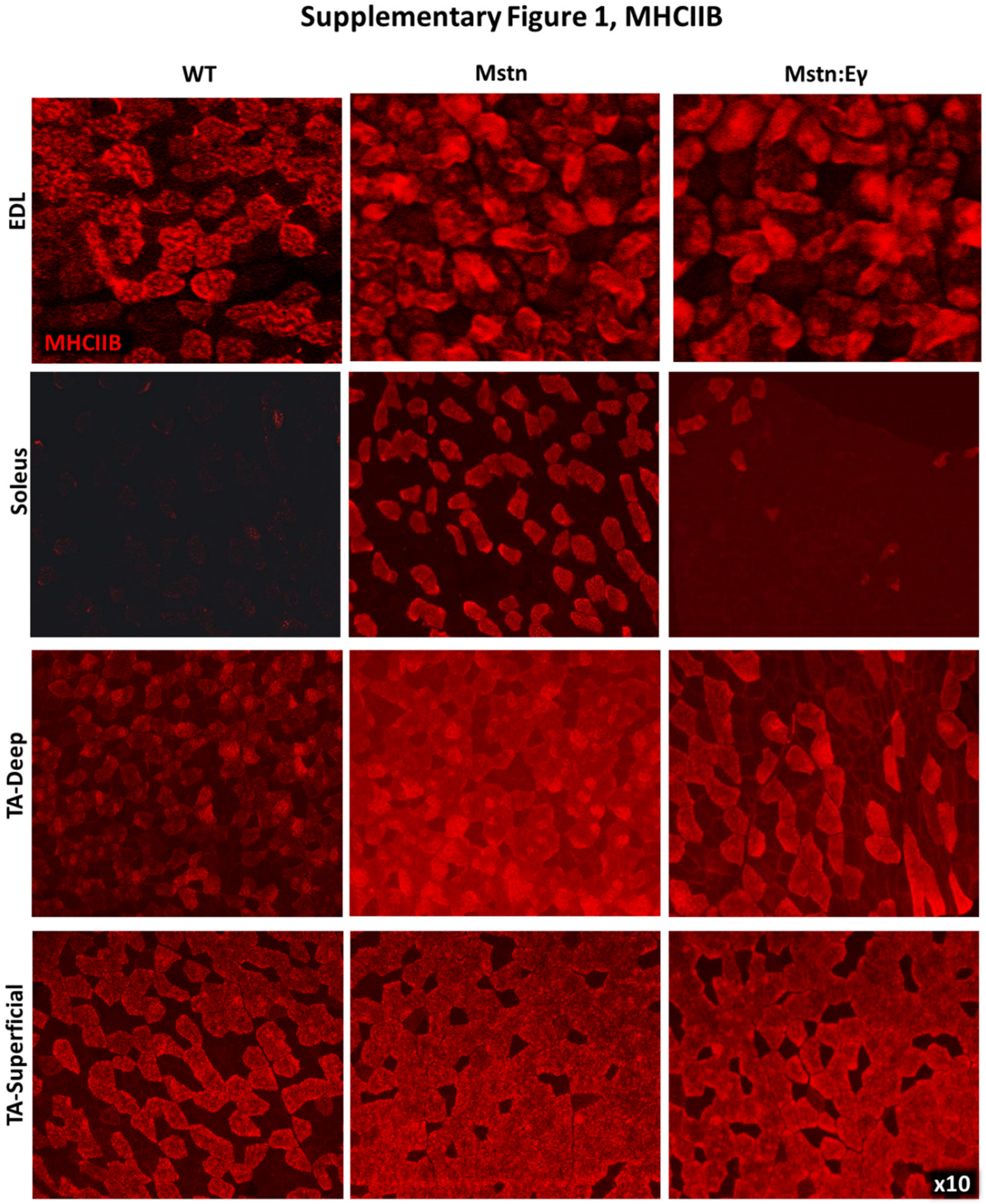

**Supplementary Figure 2.** MHCIIA profiles in WT, *Mstn*$^{-/-}$ and *Mstn*$^{-/-}$/*Errγ*$^{Tg/+}$ mouse muscle.

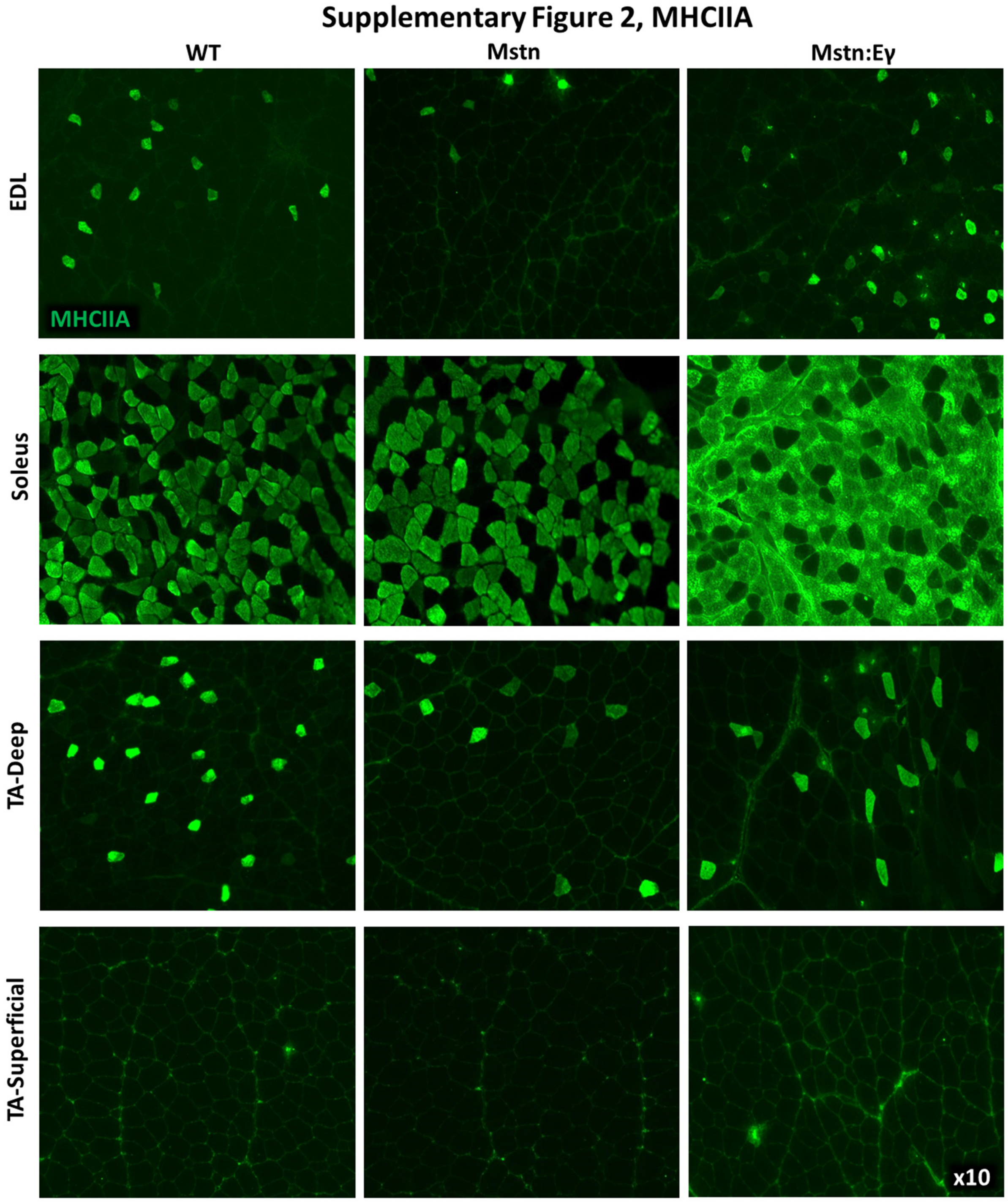

**Supplementary Figure 3.** MHCI profiles in WT, *Mstn*$^{-/-}$ and *Mstn*$^{-/-}$/*Errγ*$^{Tg/+}$ mouse muscle.

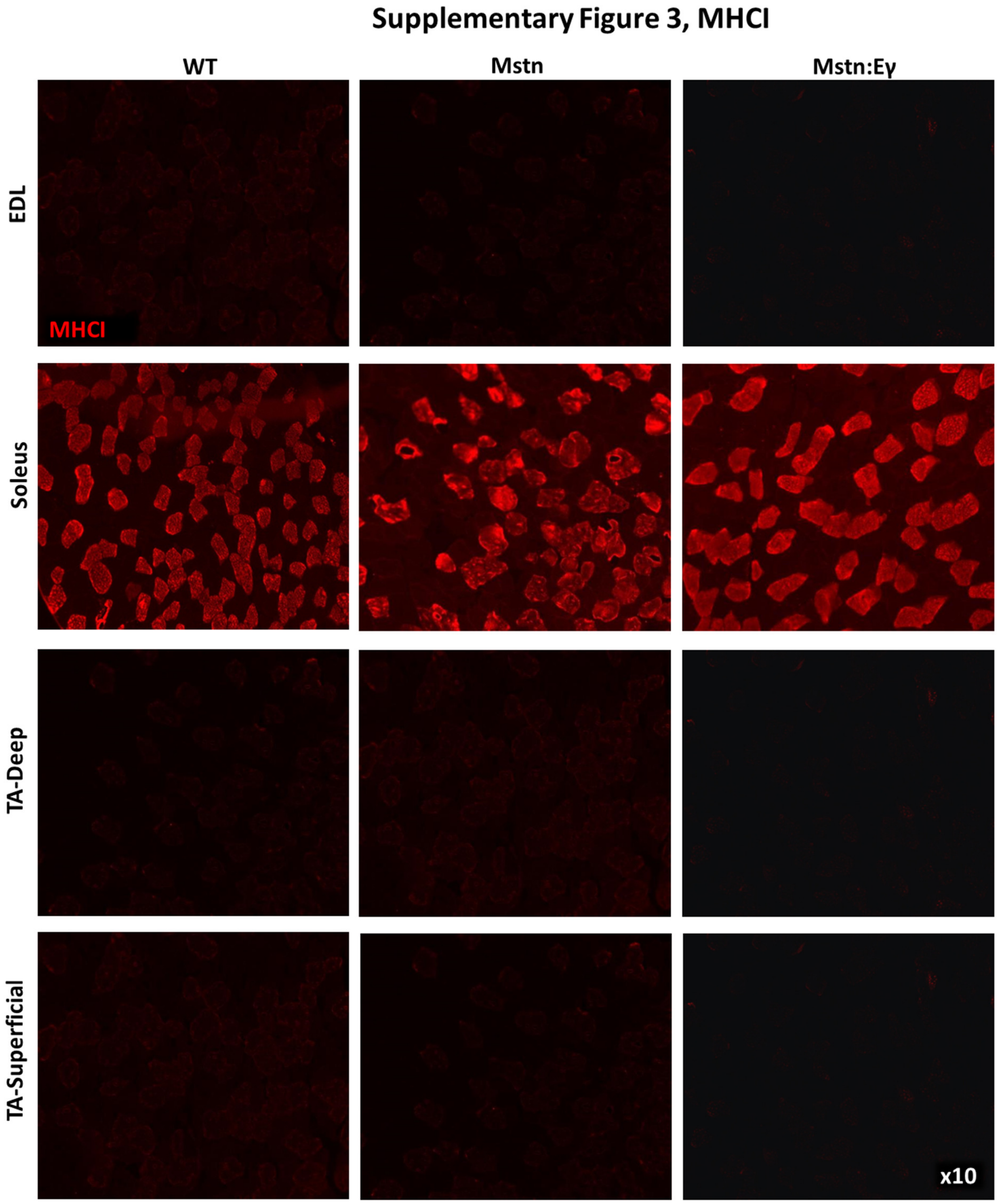

**Supplementary Figure 4.** Collagen I profiles in WT, *Mstn*$^{-/-}$ and *Mstn*$^{-/-}$/*Errγ*$^{Tg/+}$ mouse muscle. (A) Expression of Collagen I in relation to MHC fibre type. Serial section staining with MHCIIB was used to identify MHCIIB$^{-}$ (yellow stars) and MHCIIB$^{+}$ fibres (white stars). Representative MHCIIB$^{-}$ indicated with yellow stars and MHCIIB$^{+}$ fibres indicated by white stars. Expression of Collagen I more robust in ECM between two MHCIIB$^{-}$ fibres (yellow arrow) compared to that between MHCBII$^{+}$ fibres (white arrows) in wild type muscle. Same relationship albeit at lower levels in *Mstn*$^{-/-}$ muscle. Expression domain increased *Mstn*$^{-/-}$ by *Errγ* in ECM between MHCIIB$^{-}$ (yellow arrows) as well as MHCIIB$^{+}$ (white arrows) compared to *Mstn*$^{-/-}$ fibres. (B) Expression of Collagen I quantified through intensity measurements by setting standard value of 1 for the level between MHCIIB$^{-}$ fibres from WT mice. (C) Collagen I expression also quantified in terms of thick domain. n=30 from each cohort. p*< 0.05, p**< 0.01, and p***< 0.001. Statistical analysis performed by one-way ANOVA followed by Bonferroni correction of multiple comparisons.

**Supplementary Figure 4, Collagen I**

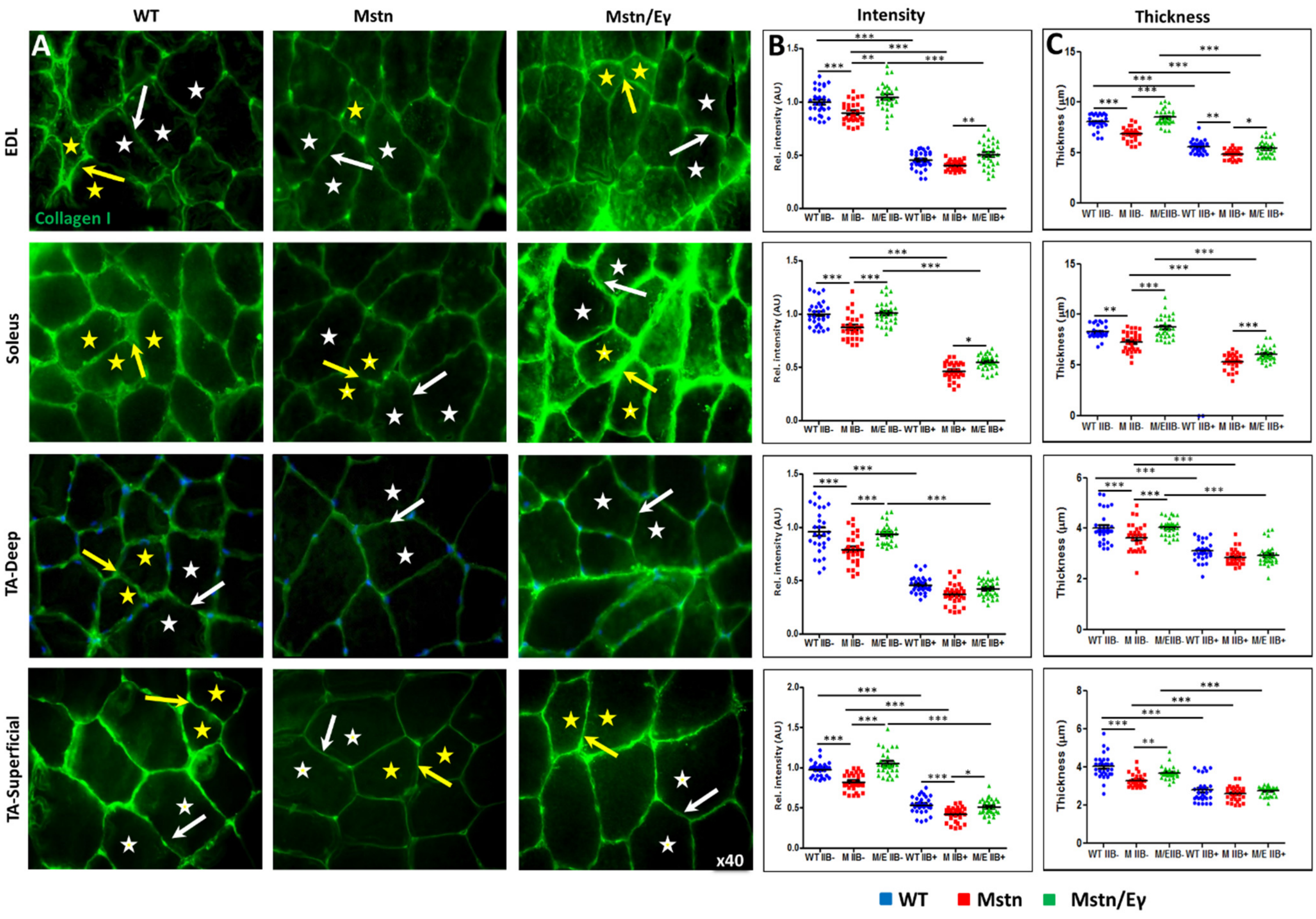

**Supplementary Figure 5.** α-Dystroglycan profiles in WT, *Mstn*$^{-/-}$ and *Mstn*$^{-/-}$/*Errγ*$^{Tg/+}$ mouse muscle. (A) Expression of α-Dystroglycan in relation to MHC fibre type. MHCIIA in green. Note higher levels of α-Dystroglycan expression in all genotypes between MHCIIB$^{-}$ compared to MHCIIB$^{+}$. (B) Expression of α-Dystroglycan quantified through intensity measurements by setting standard value of 1 for the level between MHCIIB$^{-}$ fibres from WT mice. (C) α-Dystroglycan expression also quantified in terms of thick domain. n=30 from each cohort. $p^{*} < 0.05$, $p^{**} < 0.01$, and $p^{***} < 0.001$. Statistical analysis performed by one-way ANOVA followed by Bonferroni correction of multiple comparisons.

**Supplementary Figure 5 α-DG**

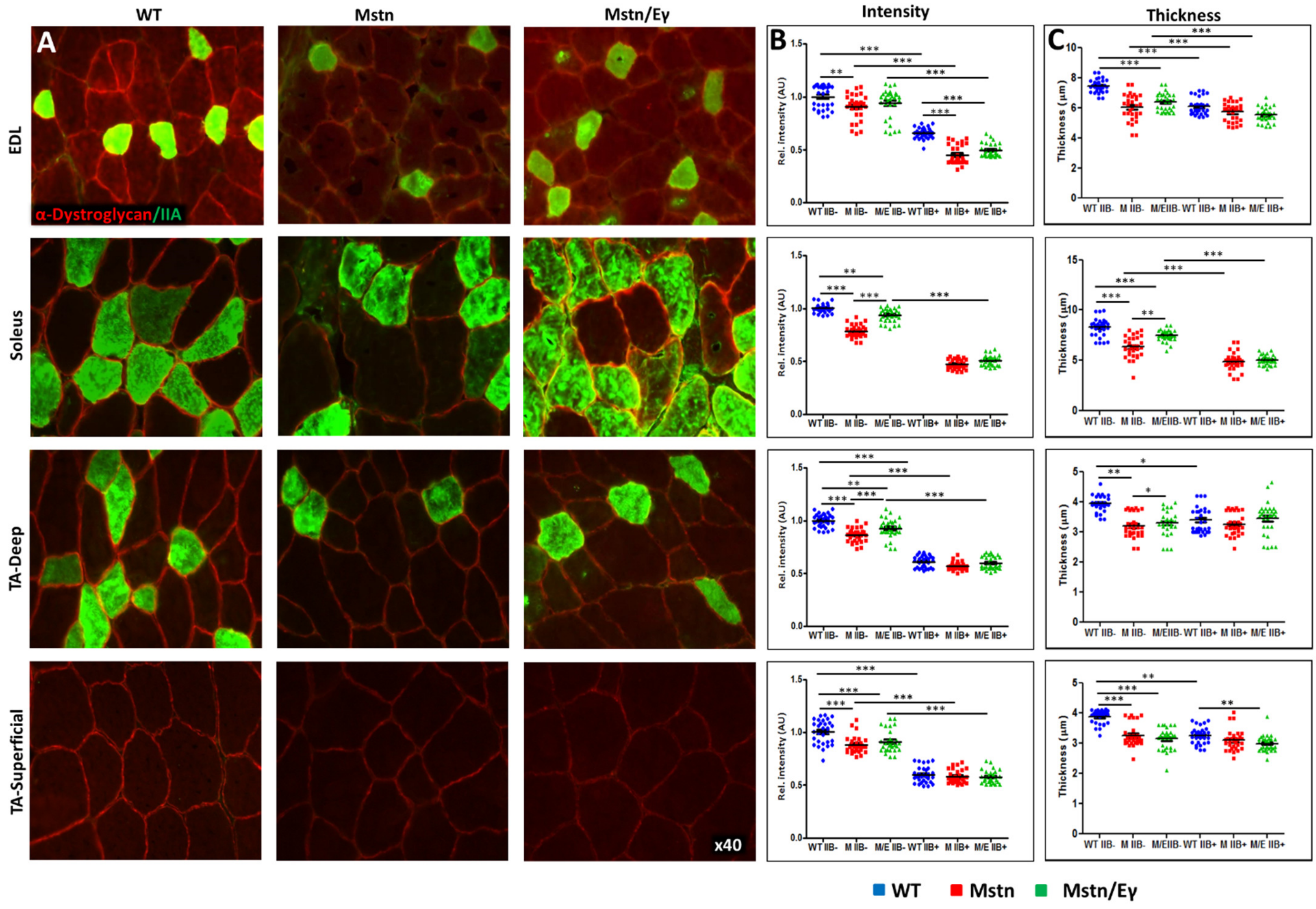

**Supplementary Figure 6.** γ-Sarcoglycan profiles in WT, *Mstn*$^{-/-}$ and *Mstn*$^{-/-}$/*Errγ*$^{Tg/+}$ mouse muscle. (A) Immunofluorescence profile of γ-Sarcoglycan in relation to MHC fibre type. MHCIIB expression in red. Representative MHCIIB$^{-}$ indicated by yellow stars and MHCIIB$^{+}$ fibres by white stars. Note higher levels of γ-Sarcoglycan in all genotypes between MHCIIB$^{-}$(yellow arrows) compared to MHCIIB$^{+}$ (white arrows). (B) Expression of γ-Sarcoglycan quantified by intensity by setting standard value of 1 for the level between MHCIIB$^{-}$ fibres from WT mice. (C) γ-Sarcoglycan expression quantification in terms of thick domain. n=30 from each cohort. p*< 0.05, p**< 0.01, and p***< 0.001. Statistical analysis performed by one-way ANOVA followed by Bonferroni correction of multiple comparisons.

**Figure Supplementary 6, γ-SG**

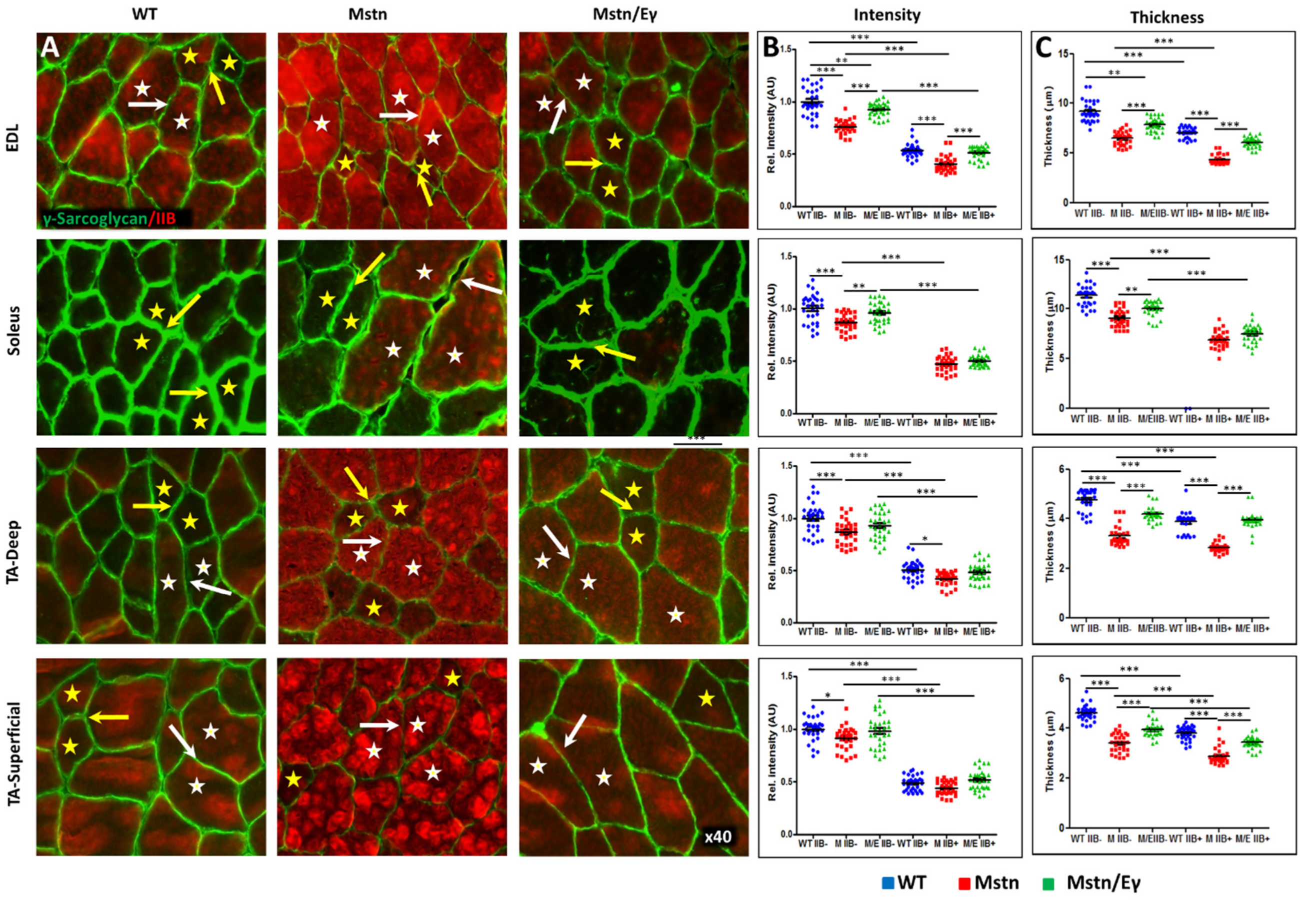

**Supplementary Figure 7.** δ-Sarcoglycan profiles in WT, *Mstn*$^{-/-}$and *Mstn*$^{-/-}$/*Errγ*$^{Tg/+}$ mouse muscle. (A) Immunofluorescence profile of δ-Sarcoglycan in relation to MHC fibre type. MHCIIB expression in red. Representative MHCIIB$^{-}$ indicated by yellow stars and MHCIIB$^{+}$ fibres by white stars. Note higher levels of δ-Sarcoglycan in all genotypes between MHCIIB$^{-}$(yellow arrows) compared to MHCIIB$^{+}$ (white arrows). (B) Expression of δ-Sarcoglycan quantified by intensity by setting standard value of 1 for the level between MHCIIB$^{-}$ fibres from WT mice. (C) δ-Sarcoglycan expression quantification in terms of thick domain. n=30 from each cohort. *< 0.05, p**< 0.01, and p***< 0.001. Statistical analysis performed by one-way ANOVA followed by Bonferroni correction of multiple comparisons.

Supplementary Figure 7 δ-SG

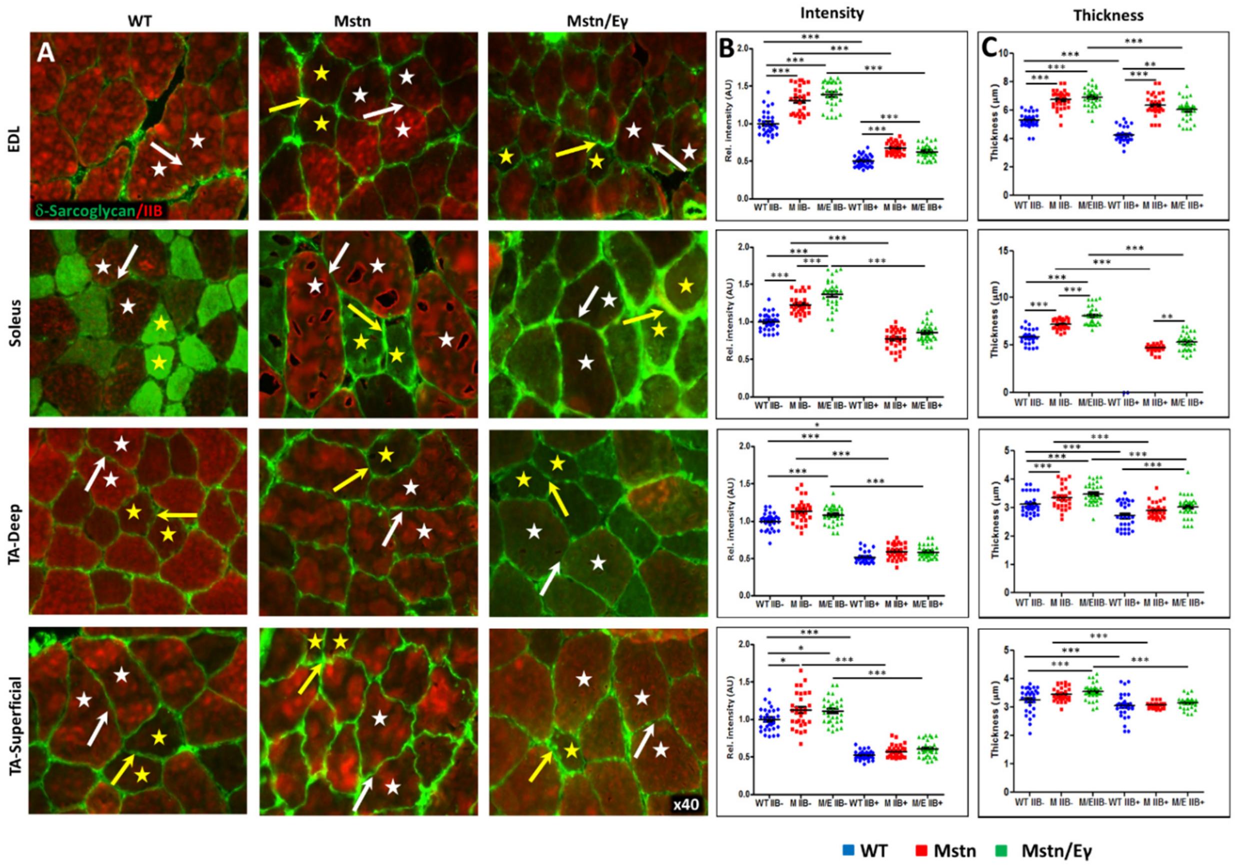

**Supplementary Figure 8 A.** Full image of the membrane probed for both dystrophin and α-actinin shown in Figure 7b. **B.** Images of the membrane strips used in Figure 7b. Overlay of Chemiluminescence image of protein bands and bright field picture of the labelled membrane to show position of protein bands relative to the molecular weight markers. Pen markings were made after transfer and upon staining with Ponceau Red S. They indicate the membrane identification code (S3) and the molecular weight positions. **C**. Validation of the specificity of the antibody to β-SG on muscle protein extracts from wild type mice (WT, positive control), *mdx* mice that lack dystrophin (MDX, negative control), and *mdx* mice over-expressing micro-dystrophin which restores expression of the sarcoglycan complex (μDYS, positive control). The 50kDa band is likely a cross-reactive band as it is not present in all samples and it does not increase in intensity with over-expression of micro-dystrophin.

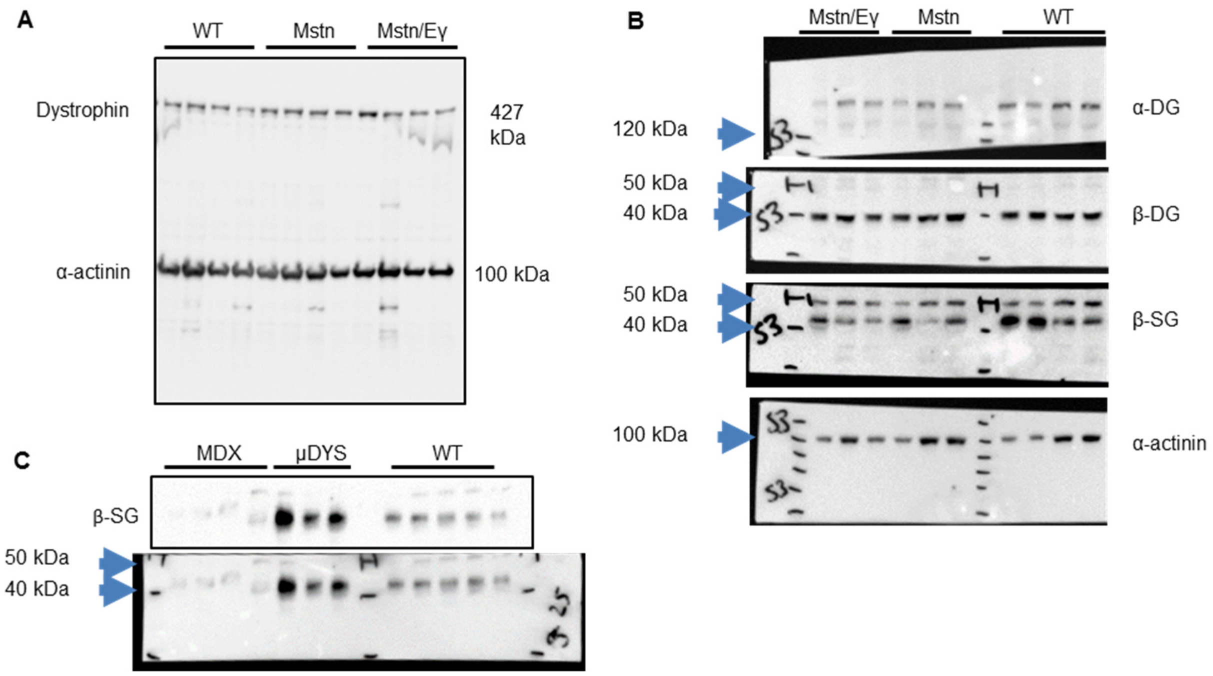

**Primary antibodies for Immunohistochemistry**

Details of antibodies and references alluding to their specificity and qPCR primer sequences.

| Antigen | Type | Immunoglo bulin | Species | Dilutio n | Supplier |
|---|---|---|---|---|---|
| **MYHCIIA** | Monoclonal | IgG | Mouse | 1:1 | DSHB A4.74 |
| **MYHCIIB** | Monoclonal | IgM | Mouse | 1:1 | DSHB BF.F3 |
| **Collagen type I** | Monoclonal | IgG | Mouse | 1:500 | Abcam |
| **Collagen type IV** | Polyclonal | IgG | Rabbit | 1:500 | Abcam |
| **Dystrophin** | Polyclonal | IgG | Rabbit | 1:200 | Abcam |
| **α-SG** | Monoclonal | IgG | Mouse | 1:40 | Leica Biosystems |
| **β-SG** | Monoclonal | IgG | Mouse | 1:50 | Leica Biosystems |
| γ-SG | Monoclonal | IgG | Mouse | 1:30 | Leica Biosystems |
| δ-SG | Monoclonal | IgG | Mouse | 1:25 | Leica Biosystems |
| **α-DG** | Monoclonal | IgM | Mouse | 1:50 | Millipore |
| **β-DG** | Monoclonal | IgG | Mouse | 1:10 | Leica Biosystems |
| **Laminin** | Polyclonal | IgG | Rabbit | 1:200 | Sigma L9393 |

**Secondary antibodies**

| Antibody | Dilution | Species | supplier |
|---|---|---|---|
| **Alexa fluor 633 anti-mouse** | 1:200 | Goat | Life Technologies # A20146 |
| **Alexa fluor 488 anti-mouse** | 1:200 | Goat | Life Technologies # A11029 |
| **Alexa fluor 488 anti-rabbit** | 1:200 | Goat | Life Technologies # A11034 |
| **Alexa fluor 594 anti-rabbit** | 1:200 | Goat | Life Technologies # A11037 |

**Primary antibodies for Western blotting**

| Antigen | Type | MW (kDa) | Species | Dilution | Supplier | Ref. |
|---|---|---|---|---|---|---|
| **Dystrophin** | Polyclonal | 420 | rabbit | 1:200 | Abcam ab15277 | 1 |
| **α-DG** | Monoclonal (IIH6 clone) | 156 | mouse | 1:2000 | Millipore 05-593 | 2 |
| **β-SG** | Polyclonal | 45 | Rabbit | 1:200 | Abcam ab203392 | |
| **β-DG** | Monoclonal (MANDAG2) | 42-45 | Mouse | 1:500 | DSHB | 3 |
| **α-Actinin** | Monoclonal | 100kDa | mouse | 1:10000 | Sigma A7811 | 4 |

References:


1- Masubuchi N et al. (2013), Exp Anim. 62(3):211-7
2- Ervasti JM and Campbell KP (1991), Cell 66(6): 1121-31; Kanadawa M et al. (2004) Cell 117(7): 953-64; Goddeeris MM et al. (2013) Nature 503(7474):136-40.
3- Helliwell TR et al. (1994) Neuromuscul. Disord. 4(2):101-13; Johnson et al. (2013) 8(8):e73224.
4- Anthony K et al. (2014) JAMA Neurol. 71(1):32-40.


**Secondary antibodies for western blot**

| **Antibody** | **Dilution** | **supplier** |
|---|---|---|
| **Goat anti rabbit-HRP** | **1:50.000** | **Jackson ImmunoResearch 111-035-045** |
| **Goat anti mouse-HRP** | **1:50.000** | **Jackson ImmunoResearch 115-035-166** |
| **Goat anti mouse IgM-HRP** | **1:10.000** | **Millipore AP128** |

**Supplementary file 2**

**qPCR primers Sequence**

| **Primer** | **Sequence** |
|---|---|
| **mDmdF** | ACTCAGCCACCCAAAGACTG |
| **mDmdR** | TGTCTGGATAAGTGGTAGCAACA |
| **mDag1F** | CAGTGTGTTCTCTATCGAGGTCT |
| **mDag1R** | CACAGGCAGATGGCACTACC |
| **mSgcbF** | GGACCGGCTCCATAAGACTG |
| **mSgcbR** | GATGACGGCCAGGATAAACAG |
